# Use of Chipless RFID as a Passive, Printable Sensor Technology for Aerospace Strain and Temperature Monitoring

**DOI:** 10.3390/s22228681

**Published:** 2022-11-10

**Authors:** Kevin Mc Gee, Prince Anandarajah, David Collins

**Affiliations:** 1 School of Biotechnology, Dublin City University, D09 NRT0 Dublin 9, Ireland; 2 The National Centre for Sensor Research (NCSR), Research & Engineering Building, Dublin City University, D09 NRT0 Dublin 9, Ireland; 3 Photonics Systems and Sensing Laboratory, School of Electronic Engineering, Dublin City University, D09 NRT0 Dublin 9, Ireland

**Keywords:** chipless RFID sensors, temperature sensor, strain sensor, structural health monitoring

## Abstract

This paper was concerned with the current level of progress towards the development of chipless radio frequency identification (RFID) sensors that are capable of sensing strain and temperature. More specifically, it was interested in the possibility that the resulting devices could be used as a passive wireless structural health monitoring (SHM) sensor technology that could be printed in situ. This work contains the development and performance characterization results for both novel strain and novel temperature sensor designs with resulting sensitivities of 9.77 MHz/%ε and 0.88 MHz/°C, respectively. Furthermore, a detailed discussion on the interrogation system required to meet the relevant aerospace sensing requirements was also discussed, and several methods were explored to enhance the multi-sensor support capabilities of this technology.

## 1. Introduction

Chipless radio frequency identification (RFID) technology is an area of research that is seeing an increasing level of interest. Many applications have been outlined for its use including their use as a passive wireless sensor technology that can potentially be printed using direct-write technologies [1,2]. Over the past decade a wide variety of tag designs have been proposed, some of which encode information in either the time domain or frequency domain characteristics of the scattering response [3]. The existing tag/sensor implementations boast varying levels of read range and sensor performance along with significant variations in interrogation system design [4,5].

This work aimed to assess some of the challenges associated with using this technology for aerospace structural health monitoring (SHM). First, this work included an exploration of some of the main requirements the interrogation system needs to meet so that the desired sensor technology performance is achieved, including dataset resolution and interrogation bandwidth. Secondly, strain and temperature sensor designs were presented and characterized. This included a novel strain sensor, and further key characterization was performed on another sensor previously discussed by the authors in [6]. A highly sensitive temperature sensor was also given proof-of-concept testing and the overall spectrum and interrogation requirements for a single pair of sensors was outlined. Finally, some techniques were proposed that can reduce the current level of complexity being imposed on the reader system by placing it on other parts of the overall technology. A tag/sensor-based approach to supporting multi-sensor implementations was presented, which used a light-dependent resistor (LDR) to remotely switch the scattering contribution of a specific sensor on or off, but the resulting differential result was too low to be considered reliable. The other technique aimed to achieve greater sensor isolation through the use of appropriately orientated interrogation antennas, and it demonstrated that the spatial selectivity of the interrogation system could potentially be doubled, without the need for complicated signal processing or ranging hardware.

Overall, this paper takes a high-level view of the overall problem at hand and references other works where necessary to provide the relevant information on each specific topic discussed. 

### 1.1. Aerospace Sensing Requirements

Temperature sensing requirements for aerospace settings can include temperature ranges starting at −150 °C and exceeding 1000 °C [7,8,9]. The range of interest is application-specific, as other scenarios may only require temperature operation from −50 °C to +125 °C [10], or −145 °C to 470 °C [11]. Resolution requirements are more difficult to quantify, but the value of at least 0.1 °C has been found in some relevant works [11]. Similarly, strain sensing will need to support strains of at least ±0.3%, as that is what basic conventional foil gauges can readily support [12]. Furthermore, foil gauge and fiber Bragg grating (FBG) technologies can support sensing with accuracies below 10 µε [13]. Said limit has also been outlined in [11] as a requirement for being capable of detecting critical cracks in aerospace structures. Vibration can also be expected in various aerospace systems with frequencies of over 1 kHz to be expected in certain situations [14,15,16]. These are merely a rough guide for the general requirements for aerospace SHM sensors but should serve as a basic set of criteria for the resulting sensors to meet. Another key criterion to this discussion is the total number of sensors required for a generic aerospace SHM application, which has been estimated to be in the order of 10^3^ [8] to 10^4^ [17] depending on the specific application. Also of interest to this discussion is a recent application to tender from the European Space Agency (ESA), seeking the development of an in situ fabrication system for the automated deployment of strain and temperature sensors onto satellite hardware [18].

In terms of the environmental challenges posed by the aerospace setting, extremes of temperature and radiation can be expected, as well as variations in other stimuli such as humidity, pressure and atmospheric constituents. More thorough discussions on the challenges posed by temperature and radiation to semiconductor-based electronics can be found in [19,20,21]. Other effects such as outgassing [22,23], the impact of atomic oxygen [24,25] and electrostatic charging [17,26] have been discussed in the referenced works.

### 1.2. Strain Sensing

A wide variety of chipless RFID strain sensors have been developed over the past number of years, including those published by the authors in references [6,27]. These recent publications (published within the last 12 months) collectively include a thorough comparison of the existing designs within the literature, so this feature will be omitted from this document.

### 1.3. Temperature Sensing

Conventional temperature sensing is conducted through the use of three commonly found sensors; resistive temperature detectors (RTDs), thermistors and thermocouples. RTDs are made from metals such as tungsten, copper and nickel, although platinum (Pt) is most commonly used [28]. Temperature ranges of over 1000 °C can be supported [29], and these devices, especially platinum-based ones, have a reasonably linear resistance change with a change in temperature [28]. With that being said, the change in resistance is usually quite small, i.e., a Pt100 grade RTD has a low-temperature sensitivity of 0.36 Ω/°C [28,29]. Thermistors on the other hand are semiconductor devices made from sintered oxides from the iron group of metals [28] and boast significantly higher changes in resistance with temperature variations [28,29]. With that being said, the response is highly non-linear, and the maximum operating temperature for commonly found thermistors is around 150 °C [28,30]. The two aforementioned devices exhibit temperature-dependent resistance values, but an alternative technology is that of thermocouples, which output a voltage when exposed to a thermal gradient. These devices rely on the Seebeck effect caused by applying a temperature gradient across two dissimilar metals connected at one end [31]. Sensitivities for commonly found thermocouples vary from 68 µV to 10 µV/°C, and some implementations can support temperatures up to 2300 °C [29]. Alternative sensing technologies also exist for SHM, such as FBGs, and certain implementations have been shown to be capable of operating at over 1000 °C [32], and polyimide-coated fibers can support temperatures of 300–400 °C [33].

#### Existing Chipless RFID Temperature Sensor Designs

Existing chipless RFID temperature sensor designs have been compared by the authors in [3] in 2018 and more recently in works such as that by Gilch et al. in [34]. Attempts at thermocouple integration into chipless RFIDs have also been performed by the authors in [35], but barium strontium titanate (BST) exhibits significant ambient temperature dependencies, which dominate over the effects of the weak millivolt DC input. Under more controlled test settings, subsequent testing revealed that the device exhibits sensitivities on the order of 13 kHz/mV, which is very low and requires careful control of the ambient temperature in order for the millivolt-level sensitivity to be detected. Furthermore, other designs that make use of BST or other such temperature-sensitive ceramics (i.e., barium titanate), require processing stages that involve heating the deposition to over 1000 °C [36,37,38], which makes them unsuitable for in situ deposition on a large range of materials. Similarly, low temperature co-fired ceramics (LTCCs) regularly have sintering temperature requirements exceeding 500 °C [39]. The materials that are commonly found in aerospace materials such as aluminium [40] and a wide variety of polymers [41] do not support temperatures above 750 °C and 400 °C, respectively.

### 1.4. Interrogation System Overview

**Figure 1:**
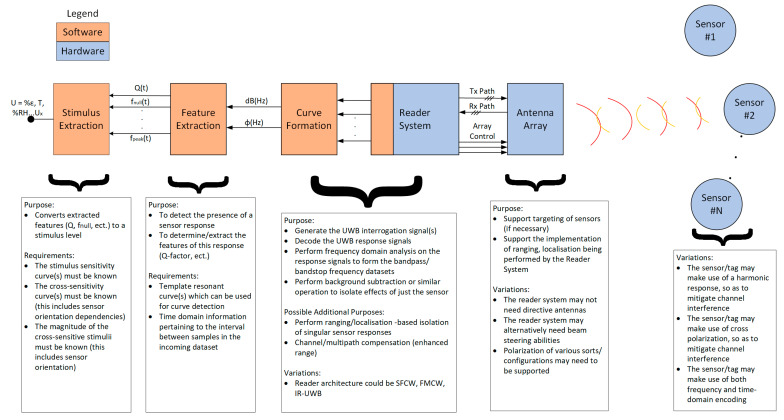
Interrogation system block diagram.

A basic diagram of the various parts of the envisaged interrogation system can be seen in Figure 1 below. This diagram includes both hardware and software components, which together allow for the stimulus (U) to be determined. The major part of this system is of course the reader system, of which several variations have been widely implemented and discussed [42,43]. These variations include: stepped-frequency continuous wave (SFCW), frequency-modulated continuous wave (FMCW) and impulse radio ultrawideband (IR-UWB) reader systems. Further detail on these systems can be found in more dedicated works such as [43,44,45]. A key comment to be made here is that much of the literature related to this topic is focused on the more widely published area of chipless RFID tag design and not that of sensor design. This is important, as only in 2020 did the sensor-related work of Marindra [46] demonstrate that some of the existing IR-UWB reader systems suffer from limited frequency resolution capabilities, with 480 datapoints being supplied for the full 4 GHz interrogation range (8.3 MHz/step). Some other general comments on the three popular architectures can be extracted from the literature, including:

- IR-UWB architectures have very fast interrogation times [42] and are highly suited to interrogating time domain tags, but they can be used with other tag types [46]. The interrogation pulse can also be of a significant power whilst maintaining UWB compliance [43];
- SFCW architectures step through each interrogation frequency and are quite slow compared to IR-UWB but allow for high levels of frequency resolution [43];
- FMCW is similar in its performance to SFCW but has intrinsic ranging capabilities [43].

#### 1.4.1. Well-Known Interrogation System Blocks

Certain blocks/steps within Figure 1 were not covered in great detail in this paper as they are somewhat well-known already. One such step is curve formation, which varies depending on the reader architecture used and performs the relevant signal processing (antenna mode extraction, background subtraction, etc.) and outputs the frequency response of the response signals. Another important step is stimulus extraction, which makes use of the known sensitivity curve along with all other stimulus information to account for cross-sensitivities, etc., and to output the final stimulus information. Also omitted from this introduction was a discussion on the use of more complex receivers such as a rake system [47], whose operation is supported by the blocks that proceed after the feature extraction block.

The sensor nodes themselves can have different design features so that the overall system can achieve a greater level of application suitability. Variations of note within the sensor design include the use of harmonic responses [48,49] or polarization diversity [50]. This work will present a novel design feature that will allow for a chipless RFID tag to be switched on/off remotely, so that its response can be isolated from other nearby tags.

Similarly, the interrogation antenna(s), referred to as the “Antenna Array” in Figure 1 below, can include omni-directional antennas or, more commonly, make use of highly directive ones. A wide variety of publications exist in this area on the topic of antenna design, but very few on the manner in which they can be used. This work will explore one way in which the interrogation antennas can be configured so as to enhance multi-sensor support.

#### 1.4.2. Feature Extraction Block

In the context of high-resolution sensing in challenging environments, a very important step is that of feature extraction. This step is often overlooked but will be of significant importance in the use of chipless RFID as a sensor technology. The choice of reader architecture has a direct impact on the challenges posed within this step, as IR-UWB systems would appear to have very limited frequency resolution [43,46], and SFCW and FMCW systems have very long interrogation times (10 ms–10 s [43]). The topic of interrogation time is only mentioned in passing here, and the manner in which sensor ranging is performed may reduce its impact, but the interrogation rate for other aerospace SHM-sensing technologies can exceed 1 kHz [51,52]. The issue of interrogation time also becomes important with time-varying stimuli such as strain, as vibration frequencies in aerospace structures can exceed 1 kHz, as mentioned earlier. Even if the time domain information is not of interest to the system designer, its effect can heavily alter the measured response curves, such that standard interpretation of the curves becomes almost impossible. More details on the extraction of time domain stimulus information from chipless RFID sensor responses can be found in [35].

Simulation results on the scattering characteristics of chipless RFID tags/sensors can be seen throughout the literature, which usually depict a noise/clutter-free environment response under idealistic conditions. As well as that, most works make use of a visual inspection of the response curves to ensure that the bandstop response is actually present in the dataset before proceeding to find the index of the minimum value in the dataset. The results gathered from many real-world test setups can include spurious dips or indeed no bandstop features within the response and other unwanted features around the null frequency; some examples of this can be seen in a variety of works including references [47,53,54]. This leads to the following questions:

- How can we determine that a sensor response is present in the returned dataset?
- Where is the minimum point of this sensor response if one is present?

The initial approach taken by Megahed [47] was to make use of a matched filter (correlation operation). This approach is highly suited for sensors which encode information in the null frequency of the scattering response. However, many sensors and tags within the literature appear to demonstrate that their resonant response changes shape significantly with a changing stimulus or dielectric constant variation [6,55,56,57,58]. One limitation of this approach is that the resolution of the extracted feature is directly related to the resolution of the frequency response dataset. What is needed is a way to decouple the feature extraction resolution from the frequency resolution of the dataset. One such way is through the use of curve fitting, as has been demonstrated by Aliasgari and Karamakar in [59]. Other works such as that by the authors in [35] made use of polynomial curve fitting, but its use is limited to working on bandstop curves that are symmetric about the null frequency. This is an important point as many of the response curves seen in the literature do not appear to exhibit symmetric responses about their null frequency [6,60,61,62]. As mentioned earlier, the work found in [59] has performed bandpass/bandstop curve fitting to the chipless RFID tag response. One difference between the ability to curve fit with tag responses and those from sensors is that the null frequency is, in theory, known before tag response curve fitting. Another notable finding in [59] is that the transfer function curve could not be perfectly fit to the response curves and was believed to be caused by fundamental differences between a bandpass/bandstop response and that from a resonant scatterer.

## 2. Materials and Methods

### 2.1. Strain Sensor Development and Testing

**Figure 2:**
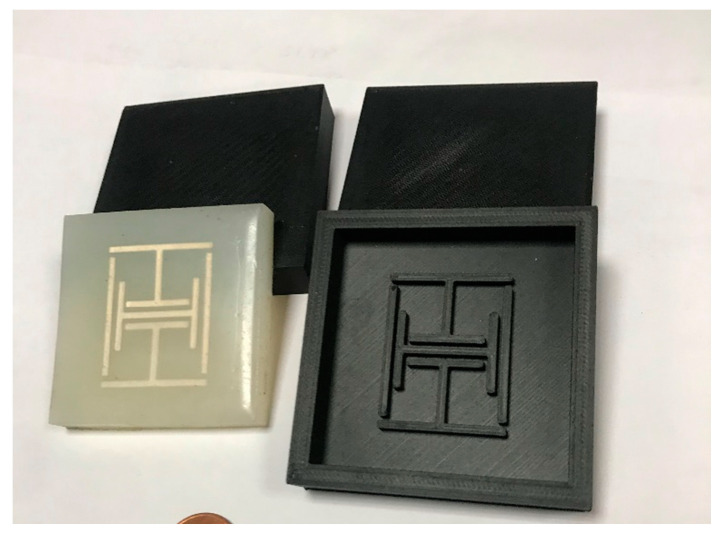
Photo of Version 3 (V3) strain sensor design and 3D-printed mold.

Two different sensors were developed and tested as part of this work. The Version 1 (V1) design was fabricated in the same way as it has been originally presented in [6]. This V3 design, which was briefly discussed during the presentation of [27], was fabricated using a different method, which made use of a molded Ecoflex™ 00–30 [63] substrate with a conductive paint-coated poly-methylmetacrylate (PMMA) resonator submerged within it. Figure 2 depicts the 3D-printed mold used and resulting sensor design. This design effectively avoids the need for thick conductors, by using a stiffer material, which performs the mechanical duties of a thick conductor. The overall dimensions of the sensor were 60 × 60 × 9 mm. The test configurations used during the testing of these designs were consistent with that used in previous publications [6,27].

### 2.2. Temperature Sensor Development and Testing

**Figure 3:**
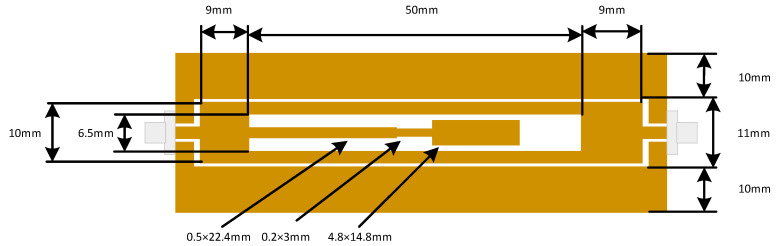
SIR circuit used in this work.

A wide variety of SHM applications will probably not require operating temperatures of over 300 °C [10] and their constituent materials are not built to operate at temperatures approaching 1000 °C, thus for temperature sensing in these types of applications, a simplified (non-ceramic based) sensor should at least be considered. Ambient temperature sensing can be achieved in a number of ways; the most simple is the use of a temperature-sensitive dielectric material, such as those outlined in [64,65,66]. More details on the origin of the temperature-dependent dielectric properties of polymers can be found in the work of Blythe and Bloor [65]. An example sensor circuit is depicted in Figure 3 below, which is based on the λ/4 stepped impedance resonator (SIR) circuits seen in the works of Amin et al. [67,68] and that by the authors in [35]. Several of these works included the relevant simulation results arising from these circuits, some of which included complex impedances attached between the end of the SIR and the ground plane.

**Figure 4:**
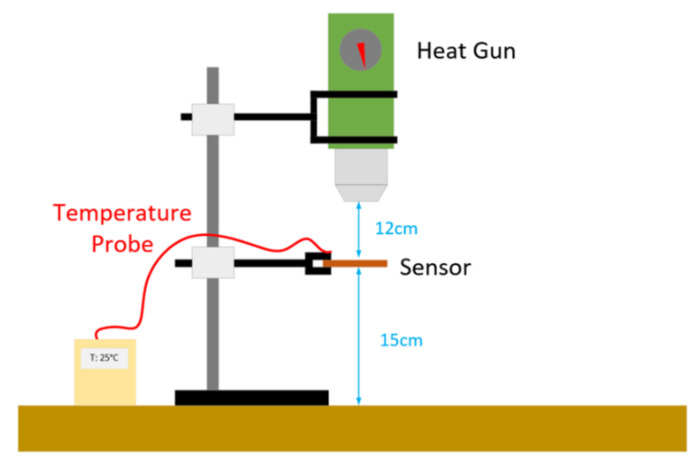
Wired sensor testing configuration.

This sensor was fabricated from a low-cost FR4 copper-clad printed circuit board (PCB) and tested in a wired test configuration at a variety of temperatures. Temperature testing was performed in a similar manner to other works [69] and consisted of a heat gun configuration, such as that depicted in Figure 4.

An alternative approach to the use of temperature-dependent dielectrics is to make use of thermally induced deformation, which could allow for larger operating temperature ranges if non-polymer materials are used [34]. This approach has been used in several works [34,70,71] with varying degrees of sensor sensitivity being achieved. The existing sensor design seen in Figure 3 was then modified to include a bi-material cantilever, similar to that seen in [71]. The resulting cantilever was made from a 300 µm thick copper sheet and a 400 µm thick PMMA layer that were adhered together using cyanoacrylate glue [72]. The cantilever was raised above the rest of the circuit with a 300 µm copper step, and the resulting assembly was soldered together. The cantilever was positioned between the ground plane and end of the SIR, similar to the approach used in [68]. Testing was once again performed using the test setup depicted in Figure 4.

### 2.3. Tag with Controllable Stimulus

**Figure 5:**
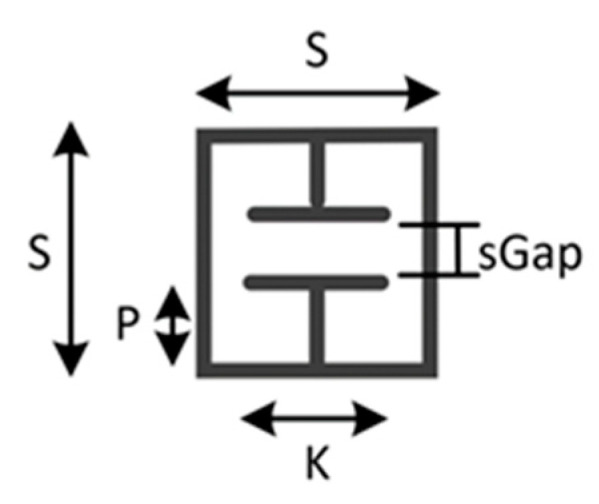
ELC resonator used in this work.

This device consisted of an FR4 ELC resonator, as detailed in Figure 5 and Table 1 below, and a cadmium sulphide LDR that was soldered between the central capacitive plates. Testing was performed using a spotlight, and luminosity measurements were made using a smartphone. The UHF/Microwave setup used was the same that used for strain sensing [6].

### 2.4. Power Distribution Testing

**Figure 6:**
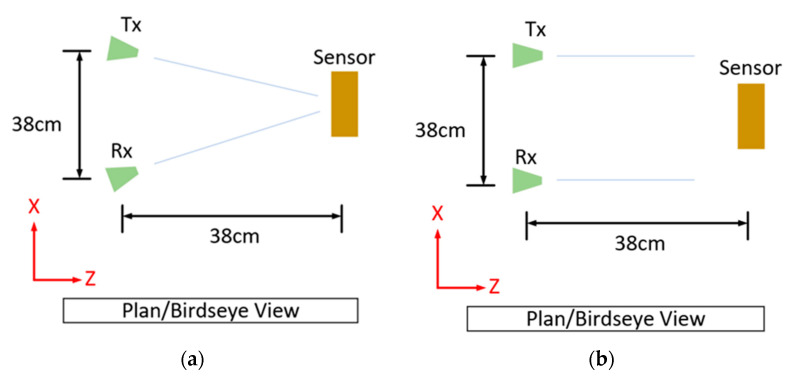
Interrogation setups: (**a**) standard and (**b**) partially illuminated.

Power distribution testing was performed with a tag (a slot resonator with a resonant frequency of 1.7 GHz), but any tag would have been sufficient. The main aspect of this testing was the two interrogation setups depicted in Figure 6 below, with interrogation distances exceeding 2λ [73] and making use of two log-periodic dipole antennas for interrogation, with a gain of 5–7 dBi.

## 3. Results and Discussion

### 3.1. Strain Sensing

**Figure 7:**
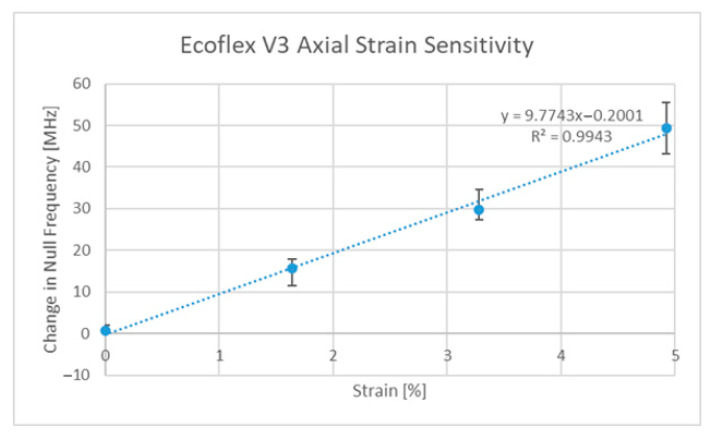
V3 axial strain sensitivity curve.

**Figure 8:**
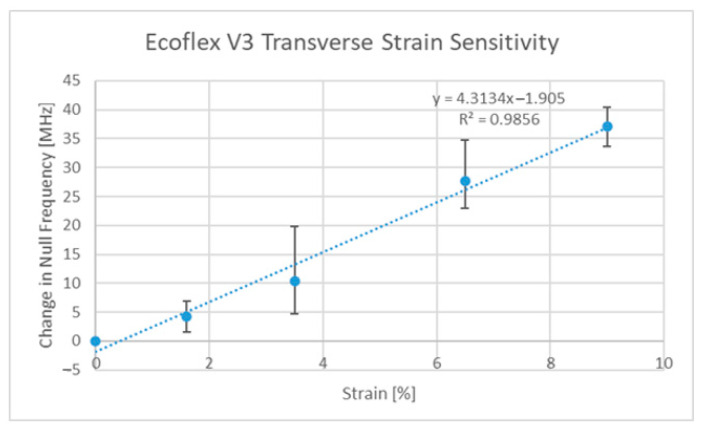
V3 transverse strain sensitivity curve.

Since the publication of two other papers on strain sensing, additional designs have been developed by the authors, such as the Ecoflex™ [63]-based implementation, which allows for strain sensing in two directions using rigid-body motion as the main deformation mechanism. The strain responses of this third version of the ELC-inspired strain gauge (V3) can be seen in Figure 7 and Figure 8 below. Said design and the V2 design [27], regardless of their implementation, still exhibit sensitivities that are lower than that of the original (V1) design [6]. Thus, the original design was the one used in all subsequent discussions. One final comment to be made at this point is that the V3 design exhibits potential cross sensitivities between the two resonances such that the current axial strain sensitivity is a function of the current level of transverse strain and vice versa. It is, however, possible to decouple the two variables if proper testing has been performed on the sensor (i.e., strain sensor testing in both directions simultaneously).

**Figure 9:**
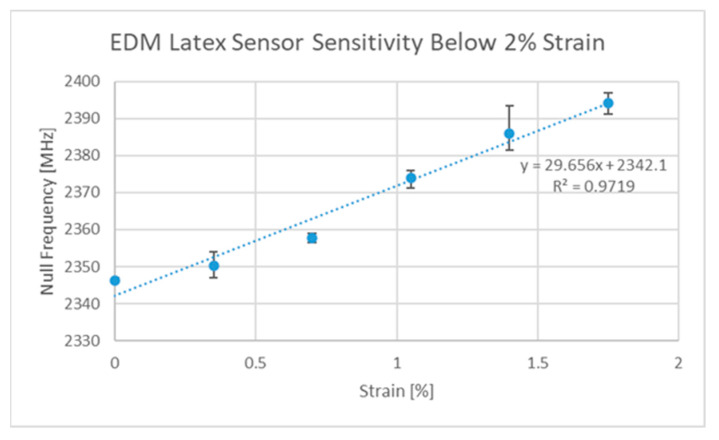
Latex V1 axial strain sensitivity curve.

**Figure 10:**
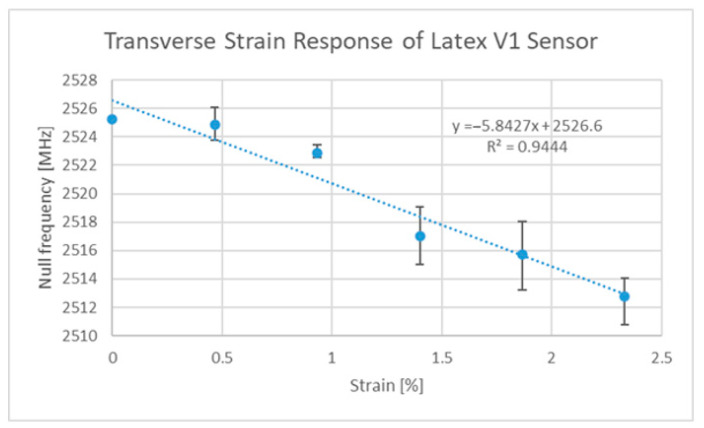
Latex V1 transverse strain cross-sensitivity curve.

The V1 design was originally published in [6] and a new version of the design is characterized in Figure 9 and Figure 10, which includes a since-unreported transverse strain sensitivity result. This new implementation was made in the same way as the previous publication except the adhesion between the resonator and the latex substrate was enhanced and was not emphasized at the centroids of the resonator parts. The result of this was a design with a slightly lower axial sensitivity of 29.7 MHz/%ε as opposed to 32.9 MHz/%ε but said changes in the adhesion resulted in a significant decrease in the transverse sensitivity (−5.8 MHz/%ε as opposed to −14.5 MHz/%ε). A subtle comment to be made at this point is that the clamping of the latex rubber did impact the response of the sensor at low strains, but additional tests with a poly-methylmetacrylate (PMMA) substrate between 0 and 0.5% strain revealed test results of similar magnitudes to that seen in Figure 9 below.

The next steps on the topic of strain sensing are to develop a rapid in situ fabrication and sensor deployment system/strategy, however this was beyond the scope of this work. This is a considerable challenge to overcome, as many of the rapid fabrication and deployment systems only support limited levels of geometric design. Several different approaches have since been tested, including thermal transfer and screen-printing approaches, but the resulting sensitivities were much lower than that seen in Figure 9 above.

### 3.2. Novel Temperature Sensor Design

**Figure 11:**
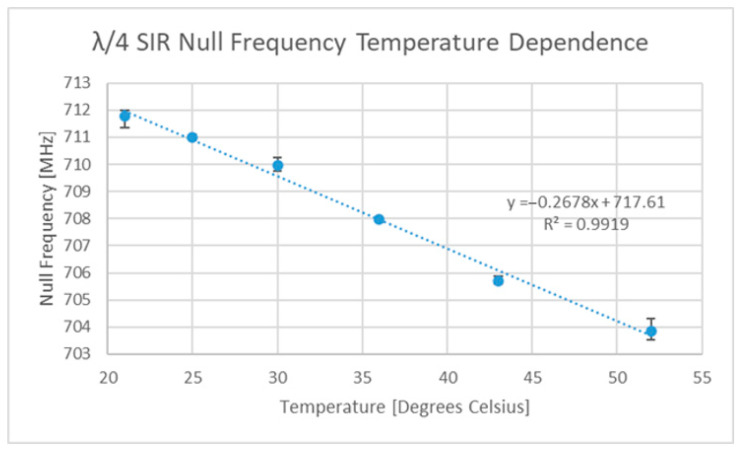
FR4 SIR circuit temperature sensitivity curve.

**Figure 12:**
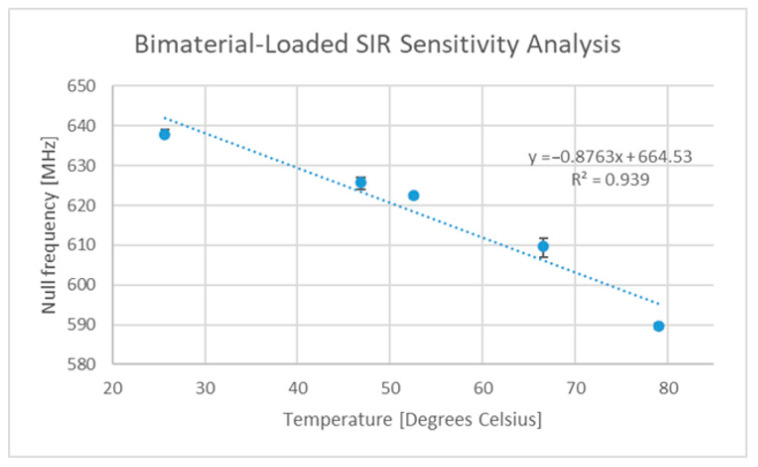
FR4 SIR circuit with cantilever temperature sensitivity curve.

The temperature sensitivity of the original FR4-based SIR device is depicted in Figure 11, which shows an average sensitivity of 267.8 kHz/°C. This sensitivity is somewhat low, but other dielectrics exist that exhibit larger temperature dependencies [64,66]. Subsequently, a bi-material cantilever was added, and the temperature tests repeated. Figure 12 depicts the temperature response of this new sensor.

**Figure 13:**
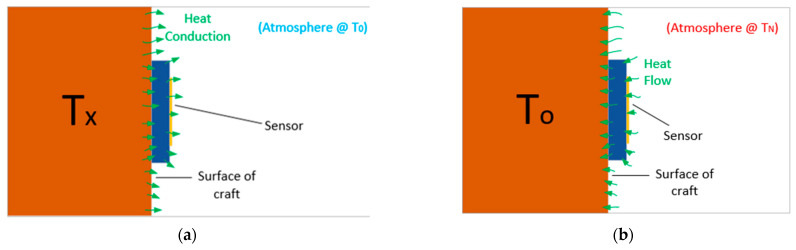
Potential heat sources: (**a**) from the surface, (**b**) from the environment.

This modified design exhibited a reasonable sensitivity of approximately 0.88 MHz/°C, which was comparable with other sensors of a similar operating frequency. One limitation exposed with this design was that the direction of the stimulus appeared to impact the level of deformation of the cantilever. Heating the sensor from different orientations other than from its back surface resulted in a more consistent sensitivity response than that found when heating from the back. This change in sensitivity went as low as 0.4 MHz/°C when the sensor was heated from the back and raised the possibility that surface and environmental temperature variations (depicted in Figure 13 below) will result in different levels of device excitation. Feeler gauges were used to assess the impact of heating the device from the top and bottom faces and revealed that the tip of the cantilever experienced deflections of 8.151 µm/°C when heated from the top and 6.952 µm/°C when heated from the bottom. Similar testing performed with the original FR4-based SIR circuit revealed no significant deviation in temperature sensitivity whether the sensor was heated from the top or bottom.

The origin of the stimulus orientation dependencies is believed to be caused by the poor heat transfer from the bottom surface over to the small cantilever element, as the large surface area of the rest of the circuit allowed large amounts of heat transfer to the environment. These types of cantilevers are used in many of the most sensitive chipless RFID temperature sensor designs, and this finding was one of considerable concern, as it may also impact said designs.

The novel design outlined above did indeed exhibit some previously undiscovered flaws, but its sensitivity when normalized against its operating frequency (gauge factor) was very high and was the second-most sensitive design found within the literature, behind a design which made use of a commercial bimetallic cantilever [70]. Further work is required to enhance the cantilever deflection and to avoid the use of PMMA within the design, as it has a glass transition temperature below 170 °C [74].

### 3.3. Survey on Sensor Interrogation

Thus far, this paper has focused on sensor design and performance, which is but a part of the overall chipless RFID sensor technology. Given the primitive nature of the chipless RFID tags in comparison to their counterparts that utilize integrated circuits (ICs), a greater amount of system complexity needs to be addressed by the reader/interrogation system. Before the topic of interrogation system design is introduced, it is important to assess what the interrogation requirements are for the developed sensors. It was also assumed that sensor deployments consisting of 10^4^ sensors cannot afford to give each sensor a unique portion of the frequency spectrum in which to operate.

Table 2 below gives a brief summary of the interrogation requirements for the sensing of strain and temperature in aerospace SHM applications. This table is somewhat geared towards the existing developed sensors, but it is important to see that the dataset size is in many cases driven by the strain sensor sensing resolution/accuracy requirements and the impact of environmental variables on the relevant dielectrics. Another important variable is the strain sensor sensitivity, as it dictates the frequency resolution requirements, much more so than that of the temperature sensor. The temperature sensor design described above has not yet been tested to the desired temperature ranges, but for the sake of this discussion, it was assumed that such a design supports the sensing of the full temperature range and that future designs in this area will achieve a similar level of sensitivity and range. The need for frequency domain sweeps of over 32000 data points is quite a challenging requirement, as the averaging of response datasets is a common subsequent operation and in sensor deployments with sensor counts approaching 10^4^, and it may not be feasible to sample the response at this level of resolution. Further work is needed into assessing how this objective can be met with the current state-of-the-art in reader design and feature extraction software.

### 3.4. Impact of Sensor and Environmental Configuration

A wide variety of challenges will present themselves, depending on the nature of the environment that the technology is deployed in. This subsection focusses on highlighting the configuration variables that could alter the sensor response, namely sensor orientation and the impact of nearby materials. This discussion did not consider the impact that certain environmental stimuli may directly have on sensitivity curve, i.e., humidity variations giving rise to strain or temperature sensor response variations.

#### 3.4.1. Sensor Orientation Effects

Other than the few chipless RFID tag/sensor designs that support an arbitrary polarization, including [46,54,75,76], the linearly polarized strain sensors presented by the authors in [6,27] and in this work exhibit resonant responses that diminish in magnitude with increasing polarization mismatch. This brings forth both challenges and opportunities [53,55]. From the results seen in [53,55], it is not yet clear if future implementations of this technology will require linearly polarized tags/sensors, so that enhanced levels of tag detection can be achieved. For the sake of this conversation, it was assumed that circularly polarized interrogation antennas can be used successfully to account for any polarization mismatch and that there is no need for the use of polarization mismatch to enhance tag/sensor detection.

It can also be seen in a variety of chipless RFID sensor designs, including [6,47,54], that the resonant response will differ in magnitude with angular variations in the line of sight (LoS) vector between the tag and the observer. This is based on the fact that the peak and null radiation patterns in the cited works are not scaled versions of each other. Frequency variations have also been reported in the responses of various chipless RFID tags caused by variations in the LoS vector [6,54], although other items of the literature have suggested that it is the structural mode of some antennas that can have orientation dependencies [77]. The orientation dependencies of the V1 design have already been partially explored in [6], and since the temperature sensor outlined above is part of a transmission line circuit, this resonator is excited in a consistent manner regardless of the orientation of the incoming signal to the reception antenna. Therefore, it is assumed that the use of a calibration tag could be used to account for orientation dependencies in the reception/transmission UWB radiation patterns and thus fully account for orientation dependencies in this chipless RFID sensor type. Although the topic of sensor orientation is an important one, the authors instead focused on the development of highly sensitive sensors above other design goals because, as seen in Table 2 above, the number of measurement datapoints that are required for aerospace SHM is incredibly high. From Table 2, it can also be seen that the strain sensing requirements dominate the total interrogation requirements. Although curve fitting could help to alleviate the issue of dataset resolution, there is no proof within the literature that said approach is reliable enough to support the resolution requirements posed by aerospace SHM. Therefore, the approach taken by the authors was to address the problem at the source, i.e., focus on enhancing the sensor sensitivities so that reader systems with limited frequency resolution could more easily locate the sensor response (IR-UWB), and so that the width of the operating frequency range is large relative to the width of the sensors resonant response. This latter point means that the entire resonant curve will be seen to move more significantly due to the applied stimulus, and thus be easier to detect and locate accurately with SF/FM-CW architectures. It will not always be possible to control the width of the resonant response as material and environmental losses may alter it, but the frequency range over which the sensors can operate can be enhanced by increasing the device sensitivity. If significant orientation challenges arise within the final implementation, it would be the intention of the authors to simply couple the developed sensor designs to a transmission-line-based tag, and thus avoid that particular problem all together.

#### 3.4.2. Effects of Nearby Materials

The sensors outlined above have only been tested in the presence of dielectric materials, but other chipless RFID sensor designs have been shown resonate/operate on metallic surfaces [46,54], although the size of the metallic surface (ground plane) and strain range may be of critical importance, as is the case found with the V1 outlined above.

Of further importance is the impact of the nearby dielectrics on the sensor response and the overall sensitivity curve. Of most relevance here is the dependency of the sensor response on the dielectric properties of the underlying material (superstrate). From the discussions found in [65,78], it is clear that a wide variety of polymers have dielectric properties that are dependent on various environmental stimuli, including temperature and humidity. The V2 design presented by the authors in [27] considered this issue more thoroughly and the challenges this represents. The main problem that could arise with the use of the presented temperature sensor is that the nearby dielectrics could have dielectric constants that vary with temperature to a different degree than that of air. Under these conditions, an apparent increase or decrease in the sensor sensitivity curve can occur and, under some certain material combinations, the sensitivity curve could, in theory, completely flatten. In the case of strain or temperature sensing, there will be a need to make a reference measurement to determine the current dielectric constant effect on the sensor response. Several key issues exist with the development of a reliable reference sensor to combat the aforementioned problems:

1. In the case of polar polymers, their environmental dependencies can be highly frequency sensitive [78]. This means that the measurement made by a reference sensor operating at a different frequency may not be indicative of the dielectric properties experienced by the main sensor. Therefore, the frequency dependence of the dielectric properties of the total nearby materials may need to be known in advance of sensor operation;
2. The sensitivity of the dielectric properties of various polymers to, e.g., temperature can be positive, negative, zero and/or nonlinear, which could lead to significant variations in the temperature sensitivity curve of the sensor;
3. The response from different resonator designs seen in [27] appears to depend to differing degrees on the nearby dielectric materials. This may mean that, ideally, the reference sensor should have the same base geometry as that of the main sensor, otherwise one of said sensors may depend to a greater degree on the dielectric behavior of one portion of the dielectrics in the environment.

The solution to these particular issues was beyond the scope of this document, but their presence must be highlighted at this point for the sake of completeness. It will most likely be the case that testing will need to be completed on all relevant aerospace materials to assess potential variations in sensor performance.

### 3.5. Potential Methods of Offloading Complexity from the Reader Architecture

Considerable challenges still exist within the development of the reader system, and many works have sought to simplify its design by moving complexity from this part of the technology to another. Works of interest include those which make use of harmonic responses from the sensors/tags, which in some cases have resulted in read ranges over one meter [5]. Other designs have used simpler approaches to give each sensor a unique portion in the spectrum, so that multi-sensor support is a smaller issue, particularly for implementations with a low number of nearby sensors [79]. Even more pronounced is the exploitation of the interrogation antenna polarization, so that enhanced response detection can be achieved [50,53].

The rest of this work will focus on enhancing the potential for multi-sensor support by offloading complexity from the reader system to the antenna array and sensor blocks seen in Figure 1 earlier. Said implementations could potentially be configured with any type of tag/sensor type, whether it be time-domain-based or frequency-domain-based. With that being said, this work will demonstrate these methods on frequency domain tags only. The topic of multi-sensor support is a difficult one to approach as the distance requirements between nearby sensors will be application specific, and the issue of near-field coupling effects will need to be avoided within nearby sensors. On the topic of the spatial resolution requirements for aerospace SHM applications, one such application is discussed by Shen et al. in [80], which has strain sensor spacings of just 20 cm. Works of relevance on the topic of multi-tag/sensor support include references [45,81,82], but very few publications consider the potential that the location of the resonant response in the frequency domain could change. However, a working physical demonstration of multi-sensor support has been presented by Henry et al. in [82,83], which uses dielectric-resonator-based scatterers over read ranges exceeding several meters, using 3D radar imaging techniques along with very highly directive antennas. This seems like a very promising result, but there could still be potential trade-offs with the use of directive antennas and other such trade-offs caused by pushing the majority of the system complexity onto the reader system; most important to this discussion is the potential impact on the final response dataset resolution and final spatial resolution.

#### 3.5.1. Tags with a Controllable Stimulus

The main problem to consider in multi-sensor systems is that multiple sensors can be illuminated by the incoming EM wave. The spatial selectivity of the incoming wave decreases with distance, and it would be of benefit if the tag could be switched on/off remotely using some other means to allow only one sensor to contribute to the measured scattering response. For this to work, a stimulus is required that can be applied to the tag(s) with a greater level of spatial resolution than that of the EM wave. In the context of frequency domain chipless RFID tags, the desired effect is that the RCS response of the tag is heavily amplified or is pushed into an unused portion of the spectrum so that it can be interrogated in isolation.

**Figure 14:**
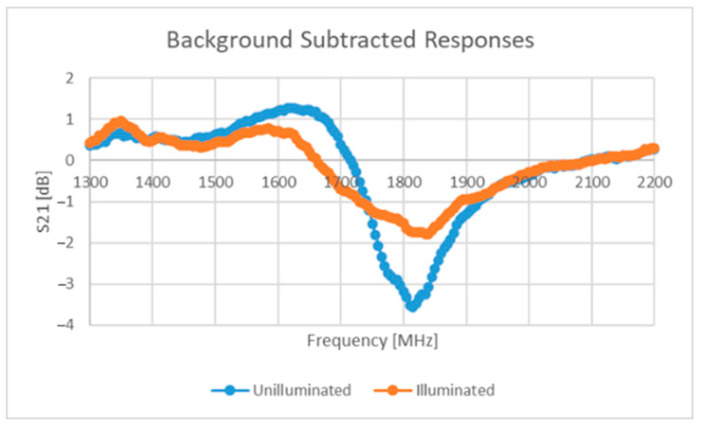
Response from illuminated and unilluminated LDR-loaded ELC tag.

**Figure 15:**
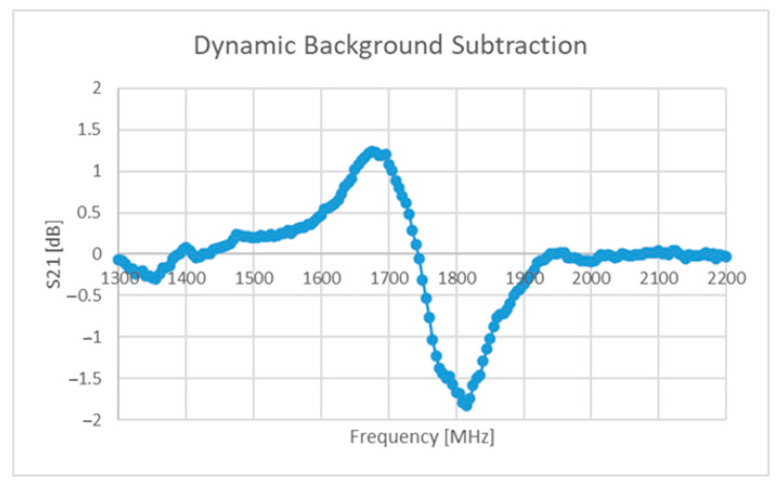
Resulting signal from subtraction operation.

One method implemented as part of this work involved the addition of a light-dependent resistor (LDR) to the capacitive region of an ELC resonator [62]. The response signals under no visible illumination and illumination at a level of 1000 LUX can be seen in Figure 14 below. The goal here is to effectively “switch off” the tag through visible illumination, which will ideally mean that the response signal is the actually the real-time background response of the environment and all of the other sensors (containing contributions from N-1 tags). Assuming that no near-field coupling is occurring between tags and that only one is being visibly illuminated, the isolated tag response can be found by subtracting the response signal found under the illumination of no tag (containing components from N tags) from the signal with contributions from N-1 tags. This result can be seen in Figure 15 below.

The performance seen above in Figure 15 is simply too weak to be considered useful and several other issues arise with the proposed solution, including:

- Cadmium sulphide (semiconductor) LDRs are sensitive to other stimuli such as temperature [84,85], which would mean that this solution is not suited for operation under a large temperature range;
- Further design is needed to ensure that a visual illumination system such as a laser or other such device can provide the necessary power to illuminate the LDR sufficiently;
- The laser device needs to have line of sight with the tag and needs to be steered appropriately;
- Appropriate filtering and/or semiconductor selection will be needed to ensure that only the laser can excite/illuminate the tag.

Leaving these limitations aside, this solution does not explicitly require highly directive antennas and could help to support the mitigation of the impact of a non-static environment. Alternative methods could be found that give tags/sensors the ability to be remotely switched on/off, but the above method was the only one found within the time spent on this work.

#### 3.5.2. Partially Overlapped Interrogation Power Distributions

**Figure 16:**
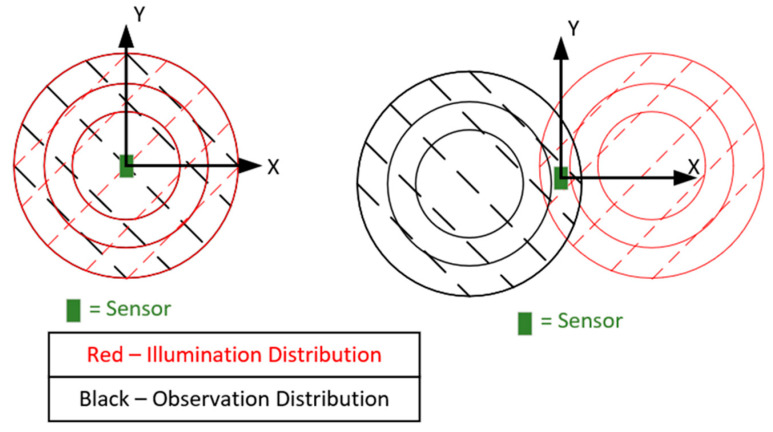
Diagram of fully and partially overlapped observation and illumination patterns.

An idea that appears in the work of Barahona et. al. in [86] is to make use of highly directive antennas in a configuration that only partially illuminates the sensor (Tx) and only partially observes (Rx) the scattering response of a chipless RFID tag. The aforementioned reference does not give any direct justification for its use, and this work has taken on this idea to explore this approach in more detail, as it may lead to a greater level of spatial selectivity. The principle behind this idea is that the spatial power distribution of the illumination wave and its observation counterpart could include multiple sensors and that their contributions to the total response is dependent on the finite directivity of these antennas. If, however, the two distributions are only partially overlapped in a certain way, then the response from a single sensor can be isolated more successfully than what can be achieved with perfectly overlapping distributions. Figure 16 below depicts these perfectly overlapped and partially overlapped distribution configurations. This approach was considered at the expense of interrogation power, as the referenced works [6,53] include results that would suggest that environmental effects play a more important role in tag/sensor detection. A critique of this approach is that it was originally conceived under the idea that single-ray propagation is the main effect within the target environment. Similar to the discussion mentioned above, the effects of near-field coupling were not considered as part of this discussion.

**Figure 17:**
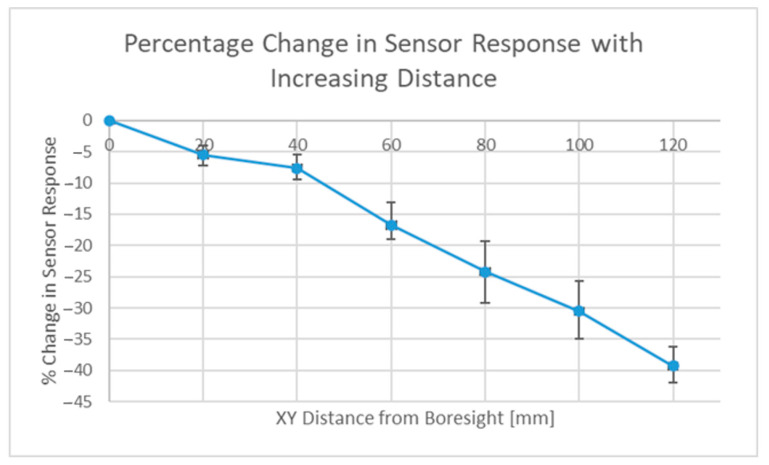
Standard (fully overlapped) distribution response magnitude results.

**Figure 18:**
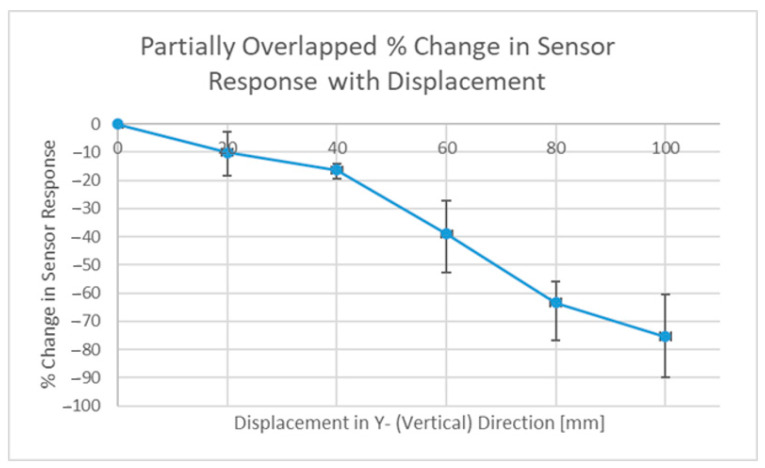
Response magnitude results from partially overlapped distribution.

The tag (a slot ring resonator) was moved vertically (Y-direction) during tests with the fully overlapped antenna configuration, and the effect that this movement had can be seen in Figure 17. Rotating the interrogation antennas into a parallel configuration revealed an alternative set of results, and these results are depicted in Figure 18 below. Note: these results will be different for different read ranges as the power distributions will be larger and potentially illuminate additional sensors (if present). Displacement in the vertical direction was tested, as movement in the horizontal plane would most likely have steeper power gradients in it because many directive antennas have sharper changes in power closer to the first null than the boresight position. With the configuration seen in Figure 16 above, the overlap of these two circles is an ellipse and the vertical direction is and always will be its major axis, regardless of how far separated the two power distributions are, thus the vertical direction would most likely be the direction in which its spatial selectivity is worst.

The results seen in Figure 17 above show that even at a distance of 12 cm, 60% of the tag response was still observed, but with the alternate setup, this was just 25%. This idea is by no means optimized, and this approach will require interrogation with beam-steering capabilities to target individual sensors and to emphasize the effect (target a smaller overlap), which may be particularly useful when the read range increases. With that being said, another benefit observed during testing was that the background measurement had a lower magnitude, as the scattering response under a single ray assumption was caused only by the region that was both illuminated and observed.

## 4. Conclusions

Overall, this paper presented a number of interesting results, including the design and testing of novel strain and temperature sensors and an initial attempt at defining the interrogation system requirements needed to make the resulting performance of the overall sensor technology suitable for aerospace applications. Along with sensor development and spectrum requirement analysis, this work also presented a thorough model of the interrogation system and subsequently discussed some of the usually overlooked aspects of its design and their importance to aerospace SHM and indeed SHM in general. A conservative viewpoint was taken throughout this work and thus some alternative approaches to combatting the challenges posed by the multi-sensor setting were also proposed and tested. These were proposed as a means of giving a future system designer additional flexibility in developing the next iteration of this technology. With that being said, future work will need to perform a more in-depth analysis of the proposed designs/solutions via mathematical and/or simulation-based modelling.

In terms of enhancing the current state of chipless RFID so that it is suitable for aerospace SHM, further work is required on all aspects of this technology. This includes but is not limited to:

- The development/testing of in situ fabrication technologies and strain sensor designs that can support the rapid deposition of a highly sensitive strain gauge onto a variety of aerospace composites;
- The development and testing of a printable, highly sensitive temperature sensor capable of operation within the entire temperature range relevant for most aerospace applications;
- The design and implementation of a full interrogation system that will enable aerospace sensing requirements to be met, including resolution, range, multi-sensor support and interrogation time requirements.

## Figures and Tables

**Table 1:** ELC geometric details.

Variable	Value [mm]	Variable	Value [mm]
S	20	Conductor Width	1.5
sGap	2	Substrate Length	40
P	6	Substrate Width	27
K	10	Substrate Thickness	1.6

**Table 2:** Spectrum requirements for sensor operation.

Performance Characteristic	Details	Required Spectrum *	Required Resolution
Strain range of ±0.25%	This range was taken as it exceeds the elastic range for metals but is below that found with many carbon- and glass-reinforced composites (CFRP and GFRP) [1]. Sensitivity of initial V1 design was approximately 30 MHz/%ε	15 MHz	Resolution/accuracy of at least 10 µε is required and the location of the null could be anywhere within the total strain sensor spectrum allocation. At a sensitivity of 30 MHz/%ε (3 kHz/µε), this resolution corresponds to 30 kHz. Assuming that the true minimum sits within ±0.5 steps of the dataset minimum, the strain sensor requires an average of 715 MHz (15 + 300 + 400), which means that the dataset will contain over 23,800 data points
Strain gauge operating temperature: −150 °C to +250 °C	This operating temperature range is not uncommon amongst some aerospace applications [3,4]. Some dielectrics can cause variations on the order of 0.5–1 MHz/°C [5]	200–400 MHz	
Detect the total strain sensor resonant urve	Detecting the whole curve allows for robust checks to ensure a valid sensor response is present [6]. The total curve ranged from 300–500 MHz	300–500 MHz	
Temperature sensor range of −150 °C to +250 °C	Previously presented sensor has sensitivity of 0.88 MHz/°C but some are on the order of 4 MHz/°C [7]	352 MHz	The dataset frequency resolution ranges from 88 kHz (0.88 MHz/°C designs) to 400 kHz (4 MHz/°C designs). The dataset required for the sensor characterized in Figure 12 is approximately 4570 datapoints in size
Detection of the entire temperature resonance curve	The total curve found in the SIR circuits seen above was less than 50 MHz	50 MHz	
Conclusion	With 715 MHz for the strain sensor and 402 MHz (average) for the temperature sensor, this leaves a total spectrum of 1117 MHz		A value of 30 kHz between datapoints is required for strain sensing and 88 kHz is required for temperature sensing. Minimum total number of datapoints should exceed 28,350

* Impact of humidity and other variables were ignored.

## Data Availability

Data available upon request and/or available on ResearchGate, accompanying details of this publication.

## Abbreviations

RFID	Radio frequency identification
SHM	Structural health monitoring
LDR	Light dependent resistor
FBG	Fiber Bragg grating
ESA	European Space Agency
PMMA	Polymethylmethacrylate
RTD	Resistance temperature detector
BST	Barium strontium titanate
SIR	Stepped impedance resonator
PCB	Printed circuit board
IC	Integrated circuit
CFRP	Carbon-fiber-reinforced plastic
GFRP	Glass-fiber-reinforced plastic
SFCW	Stepped frequency continuous wave
FMCW	Frequency-modulated continuous wave
IR-UWB	Impulse radio ultrawideband
UWB	Ultrawideband
EM	Electromagnetic
RCS	Radar cross-section
